# Rheological and Performance Research on a Regenerable Polyvinyl Alcohol Fracturing Fluid

**DOI:** 10.1371/journal.pone.0144449

**Published:** 2015-12-07

**Authors:** Xiaosen Shang, Yunhong Ding, Yonghui Wang, Lifeng Yang

**Affiliations:** 1 PetroChina Research Institute of Petroleum Exploration & Development-Langfang (RIPED-LF), Langfang, Hebei, 065007, P.R. China; 2 PetroChina Research Institute of Petroleum Exploration & Development, Beijing, 100083, P.R. China; Abdul Wali Khan university Mardan Pakistan, PAKISTAN

## Abstract

A regenerable polyvinyl alcohol/organic boron fracturing fluid system with 1.6 wt% polyvinyl alcohol (PVOH) and 1.2 wt% organic boron (OBT) was studied, and its main regeneration mechanism is the reversible cross-linking reaction between B(OH)_4_^-^ and hydroxyl groups of PVOH as the change of pH. Results of rheology evaluations show that both the apparent viscosity and the thermal stability of the fracturing fluid decreased with the regeneration number of times increasing. In addition, the apparent viscosity of the fluid which was without regeneration was more sensitive to the shear action compared with that of the fluid with regeneration once or twice. When the fracturing fluid was without regeneration, the elasticity was dominating due to the three-dimensional network structure of the formed gel; the viscosity gradually occupied the advantage when the fracturing fluid was regenerated once or twice. The settling velocity of proppant was accelerated by both the regeneration process and the increasing temperature, but it was decelerated when the proppant ratio increased. Results of core damage tests indicate that less permeability damage was caused by the PVOH/OBT fracturing fluid compared with that caused by the guar gum fracturing fluid after gel breaking.

## Introduction

Hydraulic fracturing is a favorable technique applied to the productivity improvement of oilwells located in reservoirs with the characteristics of low porosity and poor permeability. A hydraulic fracturing job consists of a series of stages, including successively injecting a preflush fluid, a proppant carrier fluid, and a displacement fluid [1]. The fracturing fluid can significantly affect the effectiveness of the fracturing. It not only determines the formation of the fractures but also affects the proppant distribution in the opened fractures. For decades, the polyacrylamide (PAM) and guar gum, or their derivatives were widely used to prepared fracturing fluids for fracturing treatments [2].

Guar gum is a kind of natural and synthetic polymer mainly constituted by the polymannose [3–5]. Despite the relatively favorable properties of guar gum-based fracturing fluid, the desired requirement of the huge amount of guar gum used for hydraulic fracturing in shale gas or tight gas reservoirs spurred the rise of its price, and it stimulated the research and development of substitutes of the guar gum. Among other kinds of fracturing fluids, polymer-based fracturing fluid was popular in China. Previously, the polymer used for fracturing was the low-cost polyacrylamide; however, its applicability in high temperature or high salinity reservoirs is unsatisfied. Moreover, the severe shear degradation of the polyacrylamide in pumps and perforations weakens its proppant carrying capacity [6]. In recent years, the viscoelastic surfactants (VES) fracturing fluid which was polymer-free was one of the hot topics. The mixture of appropriate concentrations of several kinds of surfactants replace the polymer or guar gum in VES fracturing fluid [1,7].

The three kinds of fracturing fluids (guar gum-based, polymer-based and VES-based) are most commonly-used at present. However, in the process of gel breaking and fracture clean-up, the guar gum and polymer with the high molecular weight will be irreversibly degraded and cannot be reused. Polyvinyl alcohol (PVOH) can cross-link with cross-linkers such as boron and chromium to form the fracturing fluid gel [8]. Because of the relatively low molecular weight of PVOH molecules, the gel breaking or degradation has little effect on its molecular structure; therefore, it has the potential to be reused after a proper regeneration process. Moreover, a special character of the PVOH-based fracturing fluid is that it can be regenerated as changing the pH to proper values after the gel breaking. Namely, the PVOH-based fracturing fluid can be reused under proper conditions to reduce the usage amount of the fracturing fluid, and then to save the fracturing cost. The regeneration character of the PVOH-based fracturing fluid makes it become a promising kind of fracturing fluid in oilfields. The present study described a regenerable polyvinyl alcohol/organic boron (PVOH/OBT) fracturing fluid. The regeneration mechanism of this PVOH/OBT fracturing fluid was discussed and its properties were explored in detail as follows.

## Experimental Section

### Chemicals and Fluids

Polyvinyl alcohol (PVOH 17–88, Sinopharm Chemical Reagent Company, China) with a molecular weight of 70 kDa, an effective purity of 97% and a degree of alcoholysis of 88%±2% was used as the polymer. Hydroxypropyl guar gum (HPG-1, Jingkun Chemistry Company of CNPC, China) with a molecular weight of 300 kDa and an effective purity of 96% was also used to conduct the contrast test.

The organic borate cross-linker (OBT) was self-made in laboratory using the sodium borate, glycerin, diethanolamine, sodium hydroxide and deionized water. The brief synthetic process of OBT was: (1) putting 15 wt% glycerin, 1.8 wt% sodium hydroxide and 48.2 wt% deionized water into a 3-mouth flask at the same time to prepare an even solution; (2) adding 20 wt% sodium borate and stirring the solution at 60°C for 30 min to ensure the uniform dissolution; (3) further adding 15 wt% diethanolamine into the above solution, elevating the reaction temperature to 80°C and sealing the flask for 5 h. Then the organic borate cross-linker (OBT) with a color of faint yellow and a pH value of about 9.5 was obtained.

Sodium hydroxide (NaOH) was applied to change the pH of fracturing fluids and the ammonium persulfate ((NH_4_)_2_S_2_O_8_) was used as the gel breaker. Sphere ceramic particles within a diameter range of 0.40 to 0.60 mm and an average apparent density of 2.78 g/cm^3^ were used as the proppant. Synthetic formation brine was used in experiments and its compositions are listed in Table 1.

**Table 1:** Compositions of the synthetic formation brine.

Ions	K^+^ + Na^+^	Ca^2+^	Mg^2+^	CO_3_^2-^	HCO_3_^-^	SO_4_^2-^	Cl^-^
Concentration (mg/L)	3023.5	131.3	21.5	39.8	379.6	34.1	3879.0
Total salinity (mg/L)	7508.8						

### Rheological Property Test

The rheological property tests were performed with the use of a Brookfield R/S Plus rheometer (Brookfield Engineering Laboratories, Inc. U.S.A.). In the process of shear stability tests, the apparent viscosity of each fracturing fluid sample was tested at 60°C with a shear rate of 170 1/s and a shear time from 0 to 3000 s. In the process of thermal stability tests, the apparent viscosity of each fracturing fluid sample was tested from 60°C with a shear rate of 170 1/s and a heating rate of 5°C/min. When measuring the viscoelasticity, the storage modulus G' and loss modulus G'' of each fracturing fluid system were achieved by changing the frequency scanning from 0.01 Hz to 100 Hz.

The scanning electron microscope (SU8020, Hitachi, Japan) was applied to analyze the microscopic structure of gelled fracturing fluids. The brief preparation process of the scanning sample was shown as follows: (1) uniformly smearing a small volume of gel on a frosted glass plate; (2) quickly putting the frosted glass plate into a blast dryer to remove the moisture from the gel sample; (3) putting the frosted glass plate in a vacuum coating apparatus to coat a metal film which can be conductive on the dried gel sample; (4) immigrating the frosted glass plate with the coated gel sample into the sample chamber of the scanning electron microscope. Then microscopic images can be obtained using the imaging software.

### Fracturing Fluid Preparation

In the gel breaking process, a solution with 2.0 wt% (NH_4_)_2_S_2_O_8_ was prepared as the gel breaker. The gelled fracturing fluid was broken within 1 h at 60°C after the (NH_4_)_2_S_2_O_8_ solution was added, and then the apparent viscosity of the residual fracturing fluid decreased to only 8 mPa·s. During the regeneration process, the NaOH solution (20 wt%) was used to regulate the pH from 5 to 9–10, and then the fracturing fluid gel was generally reformed. Repeated the above process and the fracturing fluid could regenerate again. In following experiments, the fracturing fluid system without regeneration was named system 1, and systems regenerated once and twice were named system 2 and system 3, respectively. After each regeneration reaction, the regenerated gel was separated with the uncross-linked fluid using a filter screen with the screen size of 75μm. The recovery ratio of the regenerated fracturing fluid system was defined as the ratio of the recovered volume of the regenerated gel to the initial volume of the system before regeneration. The recovery ratios of system 2 and system 3 were 0.77 and 0.61, respectively.

### Proppant Carrying Capacity Test

Static proppant settling tests including the single particle settling test and settling tests using different proppant ratios (the volume ratio of proppant to fluid) were performed to explore the proppant carrying capacity of fracturing fluids. To eliminate the effect of the air bubble on the proppant particles, ceramic particles were soaked in the gelled fracturing fluid at least 12 h prior to the settling tests. 1,000 mL gelled fracturing fluid was put into a transparent cylinder container (diameter 6 cm × height 60 cm) with scale in millimeter, and then soaked ceramic particles were putted into the container; meanwhile, a high-speed video device (Phantom V10, York Tech., Co., USA) was used to track particles via the middle 20 cm height of the container, and then the static settling velocity of particles was computed using the mobile image analysis software. The proppant ratios were 3, 7 and 10%, respectively.

### Core Damage Test after Gel Breaking

Four Berea cores (*ϕ* 25 mm× *L* 80 mm) with similar initial water permeability of about 60 mD were used to conduct core damage tests, and the brief experimental procedure was: (1) vacuumizing dried cores using a vacuum pump and saturating them with the synthetic formation brine; (2) injecting 5.0 PV (pore volume) the broken fracturing fluid into cores; (3) placing cores into an oven at 60°C for 2 h; (4) flooding the fracturing fluid out by inversely injecting the formation brine until the injection pressure leveled off, and measuring the final water permeabilities of cores.

The core damage ratio can be calculated using the following Eq (1).

$$F_{damage}=\frac{1-K_{final}}{K_{initial}}\times 100\%$$ (1)

where *F*_*damage*_ is the core damage ratio in percentage; *K*_*initial*_ is the initial water permeability of core in millidarcy; *K*_*final*_ is the final water permeability of core in millidarcy.

A laser particle size analyzer (GSL-101B, Liaoning Instrument Co., Ltd., China) with a measurement range of 0.02 to 2,000 μm was applied to measure the particle size distribution of the diluted fracturing fluid after gel breaking. The laser wavelength was 450 nm and the experimental temperature was25°C. The median size was calculated to characterize the distribution range of broken gel particles.

## Regeneration Mechanism

**Fig 1:**
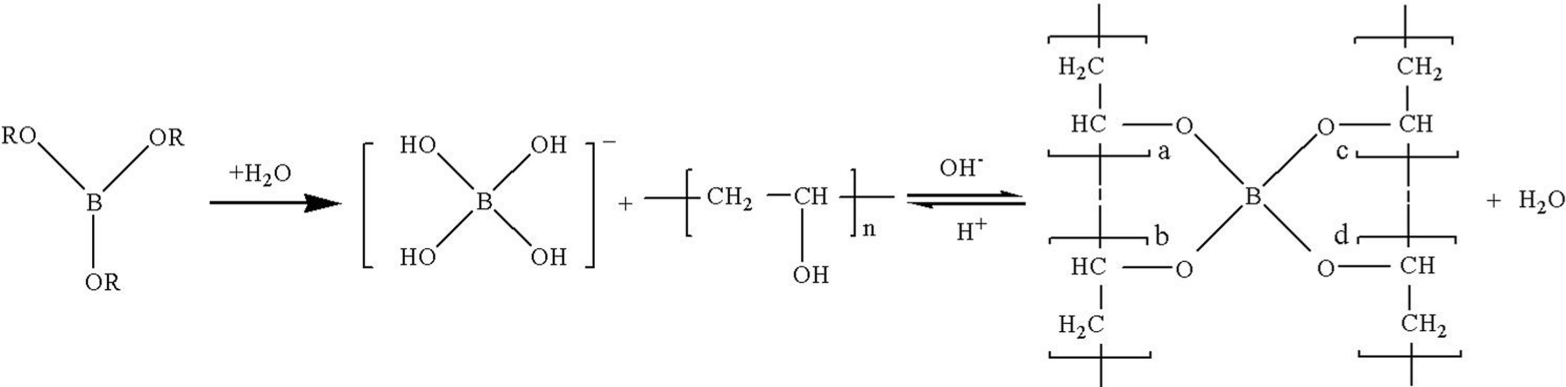
Sketch map of the cross-linking reaction mechanism of the PVOH/OBT fracturing fluid.

When the cross-linker OBT is added into the PVOH solution, the dissociation of OBT occurs in the presence of water molecules, and then acid radical ions B(OH)_4_^-^ are slowly generated [9]. B(OH)_4_^-^ can cross-link with hydroxyl groups which exist on PVOH molecular chains to form the PVOH/OBT gel system [10]. The deprotonation process of OBT and the cross-linking reaction process between B(OH)_4_^-^ and PVOH are shown in Fig 1. This chemical reaction is reversible and it is greatly affected by the acidity- alkalinity of solution. Fig 1 also indirectly proves that the acidic condition is bad for the positive reaction of PVOH and OBT cross-linking. Therefore, the extent of the cross-linking reaction between OBT and PVOH can be adjusted by changing the pH value of the fracturing fluid [11].

As mentioned above, the PVOH/OBT gel system is formed under weak alkaline condition. When the (NH_4_)_2_S_2_O_8_ is added into the fracturing fluid, the H^+^ concentration increases and it contributes to the reverse reaction shown in Fig 1, i.e., the cross-linked PVOH/OBT gel structure is easily broken under acidic conditions. In this process, PVOH molecules keep the same state as the original; therefore, the PVOH/OBT gel structure can be regenerated when the pH of solution is enhanced again [12].

## Results and Discussion

### Fracturing Fluid Screening

**Fig 2:**
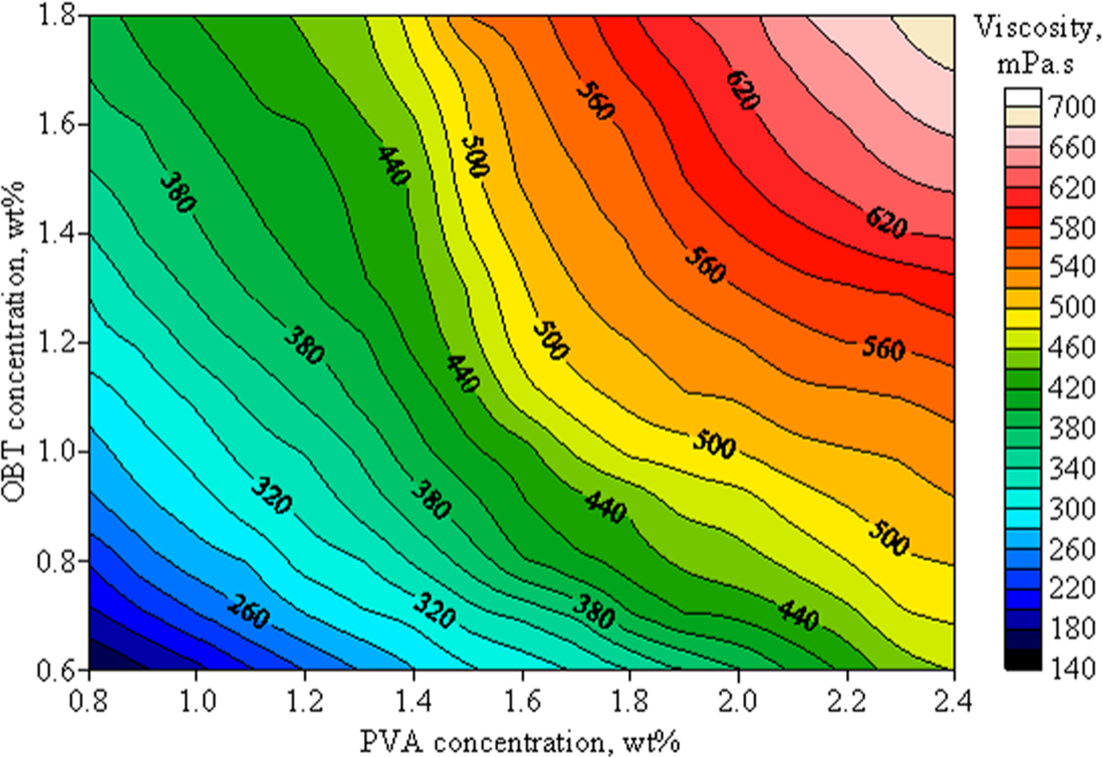
Effects of PVOH and OBT concentrations on the apparent viscosity of the fracturing fluid.

**Fig 3:**
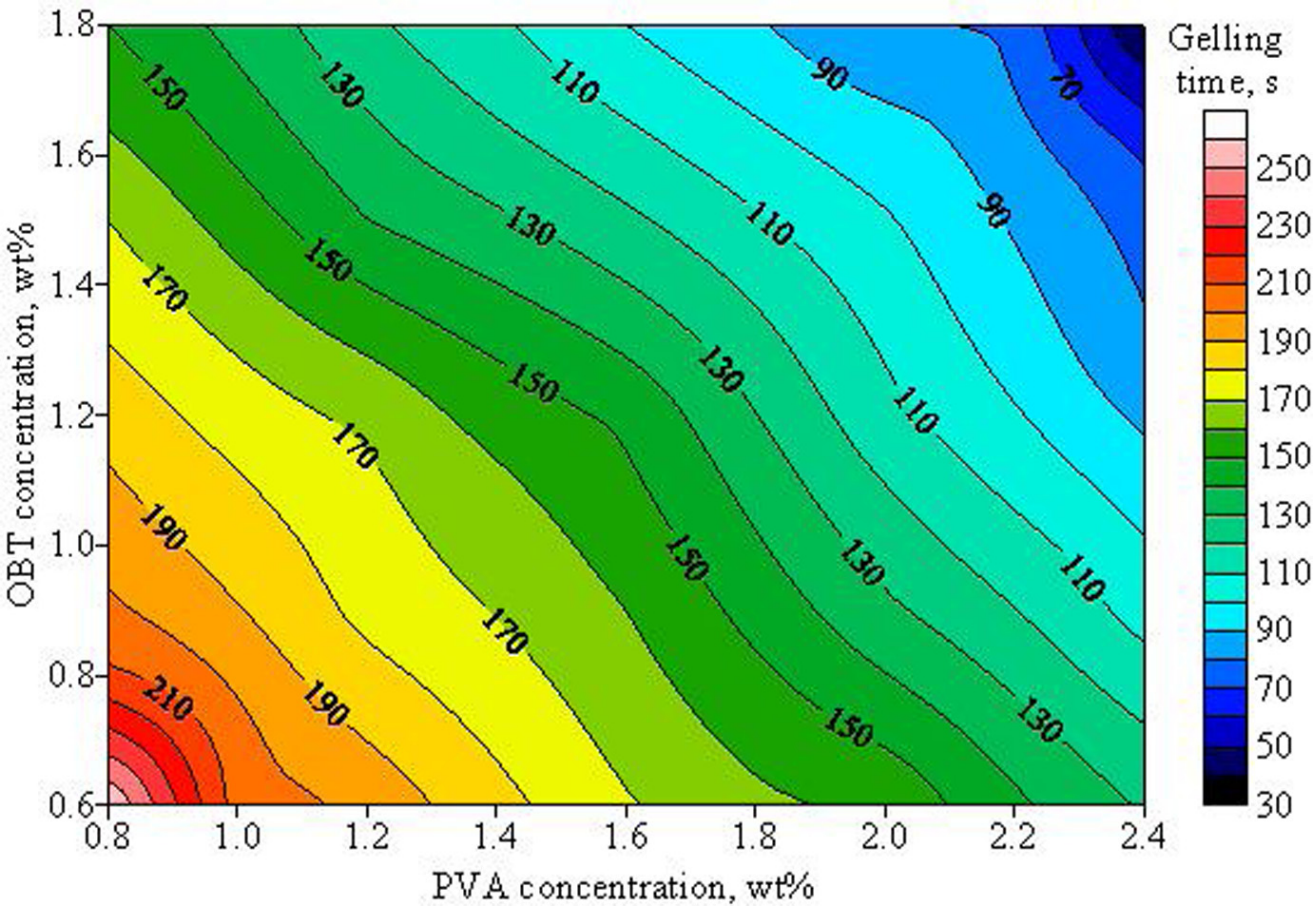
Effects of PVOH and OBT concentrations on the gelling time of the fracturing fluid.

A main purpose of this study is to develop a regenerable fracturing fluid system with favorable rheological properties and environmental-friendly performances. To achieve this goal, different concentrations of PVOH and OBT were combined to form alternative systems. After the formation of fracturing fluid gel, the apparent viscosity and the gelling time of each alternative system was measured and plotted in Figs 2 and 3, respectively.

**Fig 4:**
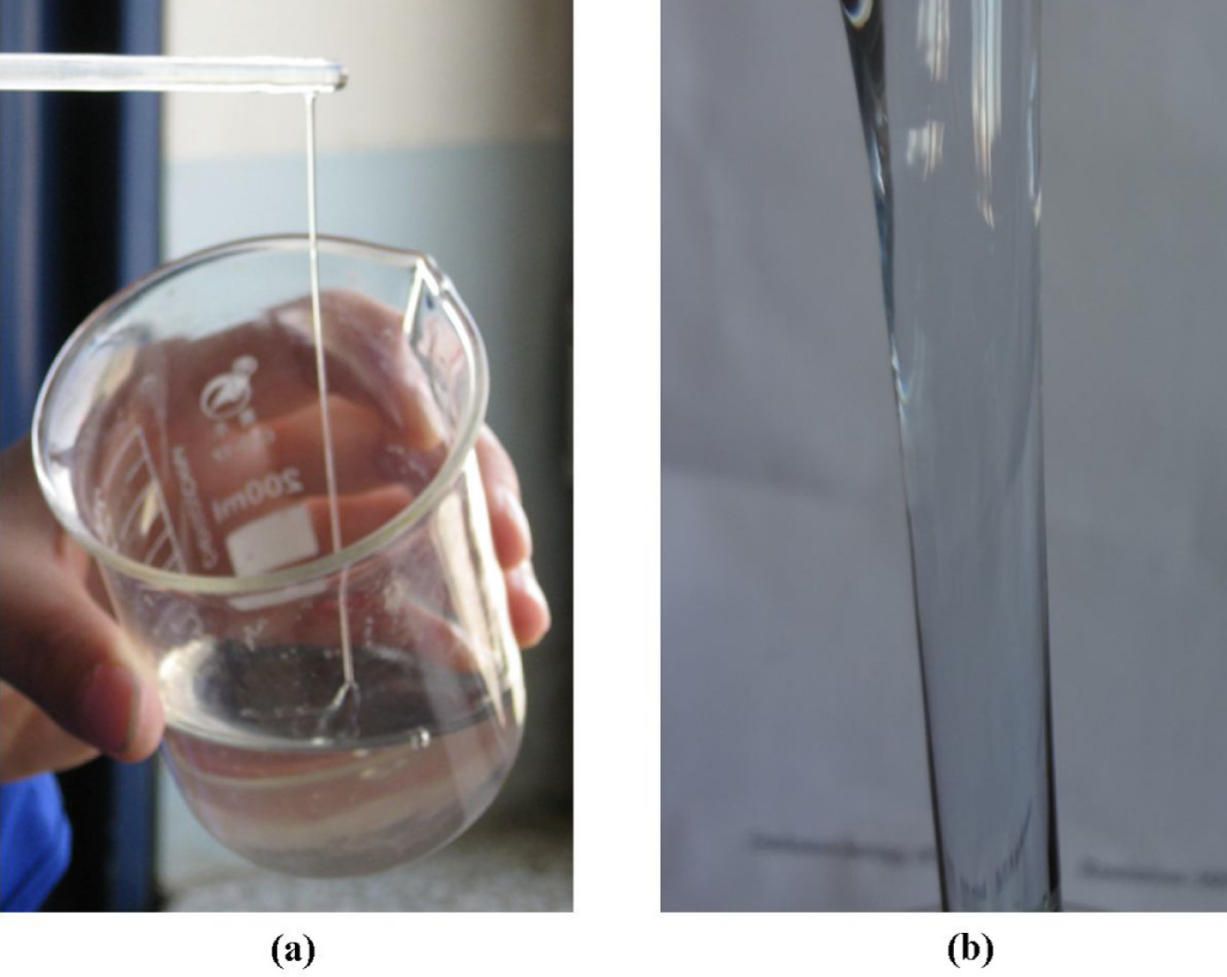
Photographs of the PVOH/OBT fracturing fluid. (a) Uncross-linked fresh fluid (1.6 wt% PVOH + 1.2 wt% OBT); (b) cross-linked gel.

Fig 2 illustrates that the apparent viscosity of cross-linked fracturing fluid was enhanced and the gelling time was shortened with PVOH and OBT concentrations increasing, as shown in Fig 3. When the concentrations of PVOH and OBT were respectively 0.8 wt% and 0.6 wt%, the apparent viscosity of the fracturing fluid gel was only 159.8 mPa·s and the gelling time was about 260 s. With the increase of PVOH and OBT concentrations to 1.6 wt% and 1.2 wt%, the apparent viscosity of gel was enhanced to 488.2 mPa·s and its gelling time decreased to 148 s. With a further increase of PVOH or OBT concentration, the apparent viscosity increased with a slow increment, but the gelling time was further reduced to the value lower than 100 s. Data indicate that the concentration of PVOH or OBT has a great effect on the cross-linking reaction between PVOH and OBT. According to the result of screening tests, the formula of the gelled PVOH/OBT fracturing fluid system which includes 1.6 wt% PVOH and 1.2 wt% OBT was selected and used in the following study. The apparent viscosity and the gelling time of this system were 488.2 mPa·s and 148 s, respectively. Photographs of the ungelled fracturing fluid and the cross-linked fracturing fluid gel were taken and shown in Fig 4.

**Fig 5:**
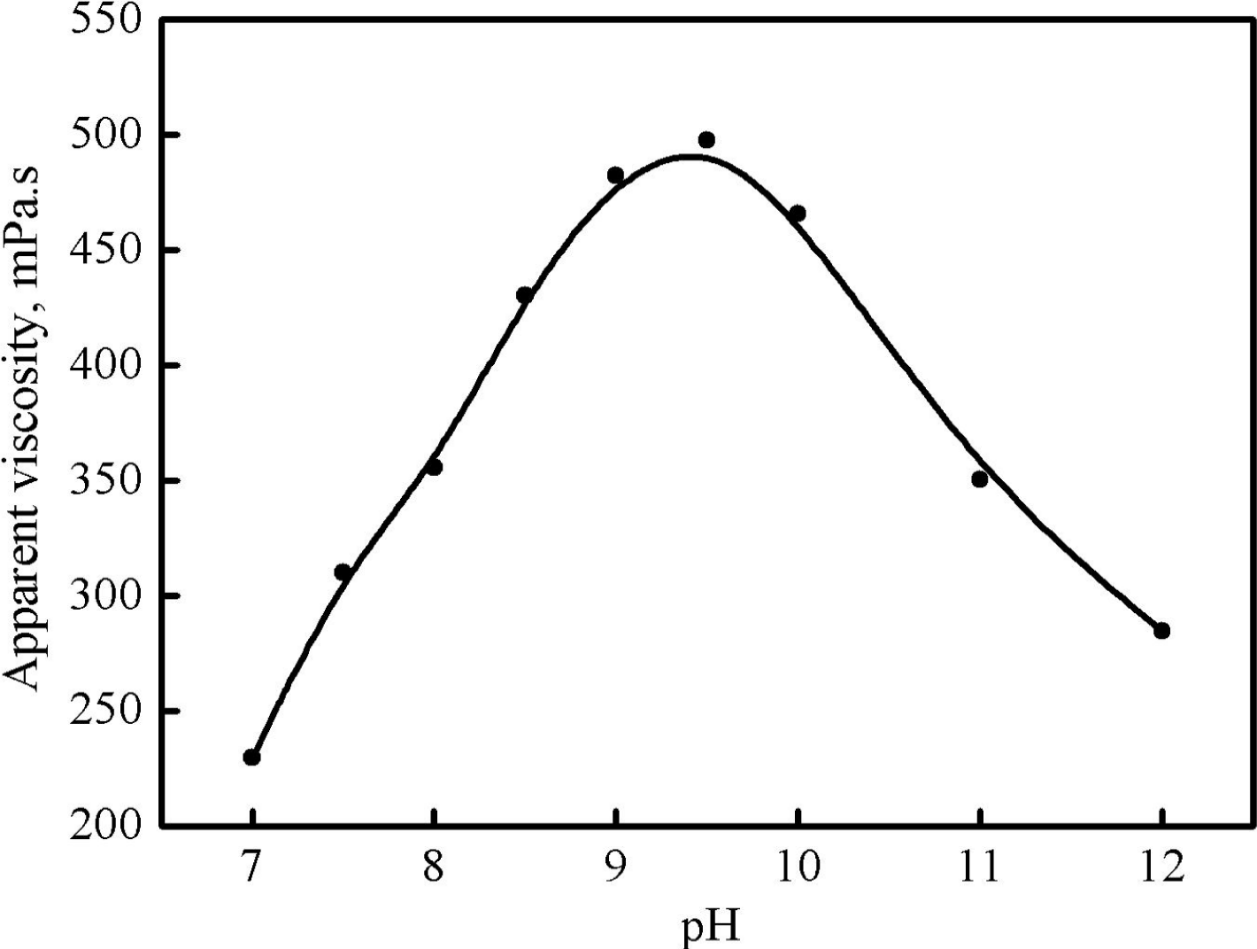
Effect of pH on the apparent viscosity of the cross-linked fracturing fluid.

The influence of pH on the apparent viscosity of the selected fracturing fluid system (1.6 wt% PVOH+1.2 wt% OBT) is shown in Fig 5. It indicates that with the increase of the pH from 7 to 12, the apparent viscosity first increased and then decreased. A peak value of the apparent viscosity (488.2 mPa·s) was obtained when pH was 9.5. Data show that the suitable pH of cross-linked fracturing fluid (viscosity>400 mPa·s) was in the range of 8.5–10. The cross-linker OBT is a kind of boric acid which does not ionize to generate H^+^ but grabs a hydroxide group OH^-^ from water molecules to yield B(OH)^4-^ and a hydrated H^+^, as shown in the Eq (2) [9]:

$$B{\left(OH\right)}_{3}+2H_{2}O\overset{\;}{\underset{\;}{⇌}}B{\left(OH\right)}_{4}^{-}+H_{3}O^{+}$$ (2)

Under the acidic condition, the above deprotonation process tends to be inversely react and B(OH)_3_ is the main mode of occurrence in the solution; therefore, the B(OH)_4_^-^ can not stably exist under strong acidic condition. Moreover, the molecules of PVOH tend to be huddle up under high alkaline conditions due to the ionic shield effect on hydroxyl groups. Therefore, there is a proper pH range for the cross-linking reaction between OBT and PVOH.

### Rheological Properties

Three fracturing fluid systems with or without regeneration were prepared for experiments: system 1 was the fresh fracturing fluid (1.6 wt% PVOH+1.2 wt% OBT) which was without regeneration; system 2 and system 3 were regenerated once and twice, respectively.

#### Microscopic structure analysis

**Fig 6:**
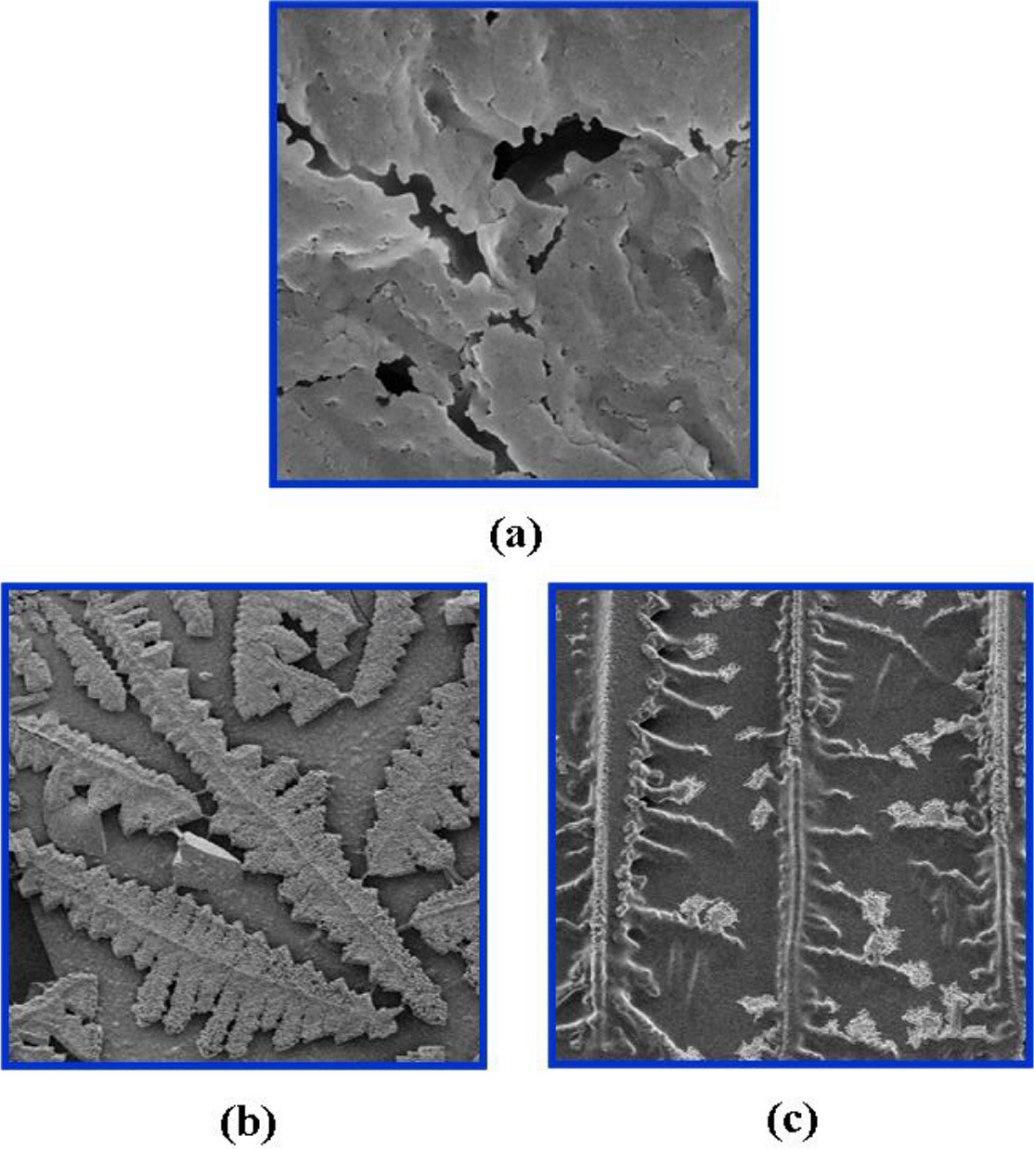
Microscopic structures of three fracturing fluid systems without or with regeneration (magnified 500 times). (a) System 1: without regeneration; (b) system 2: regenerated once; (c) system 3: regenerated twice.

Microscopic structures of three gelled fracturing fluid systems were achieved and shown in Fig 6(A), 6(B) and 6(C), respectively. Differences in microscopic skeletal structure were exhibits among three systems. As shown in Fig 6(A), the microscopic image of system 1 showed a favorable three-dimensional network structure. Fractures and cavities in Fig 6(A) were attributed to the incomplete cross-linking or the local syneresis. Syneresis is defined as the process that the gel compresses the liquid in its network structure and shrinks its volume, which is generally caused by the excessive cross-linking [13,14]. The microscopic structure of system 2 (Fig 6(B)) was like thick branches, it manifests that the extent of the cross-linking reaction of system 2 was greatly reduced when the fracturing fluid was regenerated once. The microscopic structure of system 3 was a herringbone texture and it was due to a further low extent of cross-linking reaction of system 3 compared with that of system 2. Two explanations account for the decreasing degree of cross-linking reaction between PVOH and OBT: the degradation of PVOH molecules during the gel breaking process and the contraction of PVOH molecules due to the ionic shield effect on hydroxyl groups [15].

#### Shear stability evaluation

**Fig 7:**
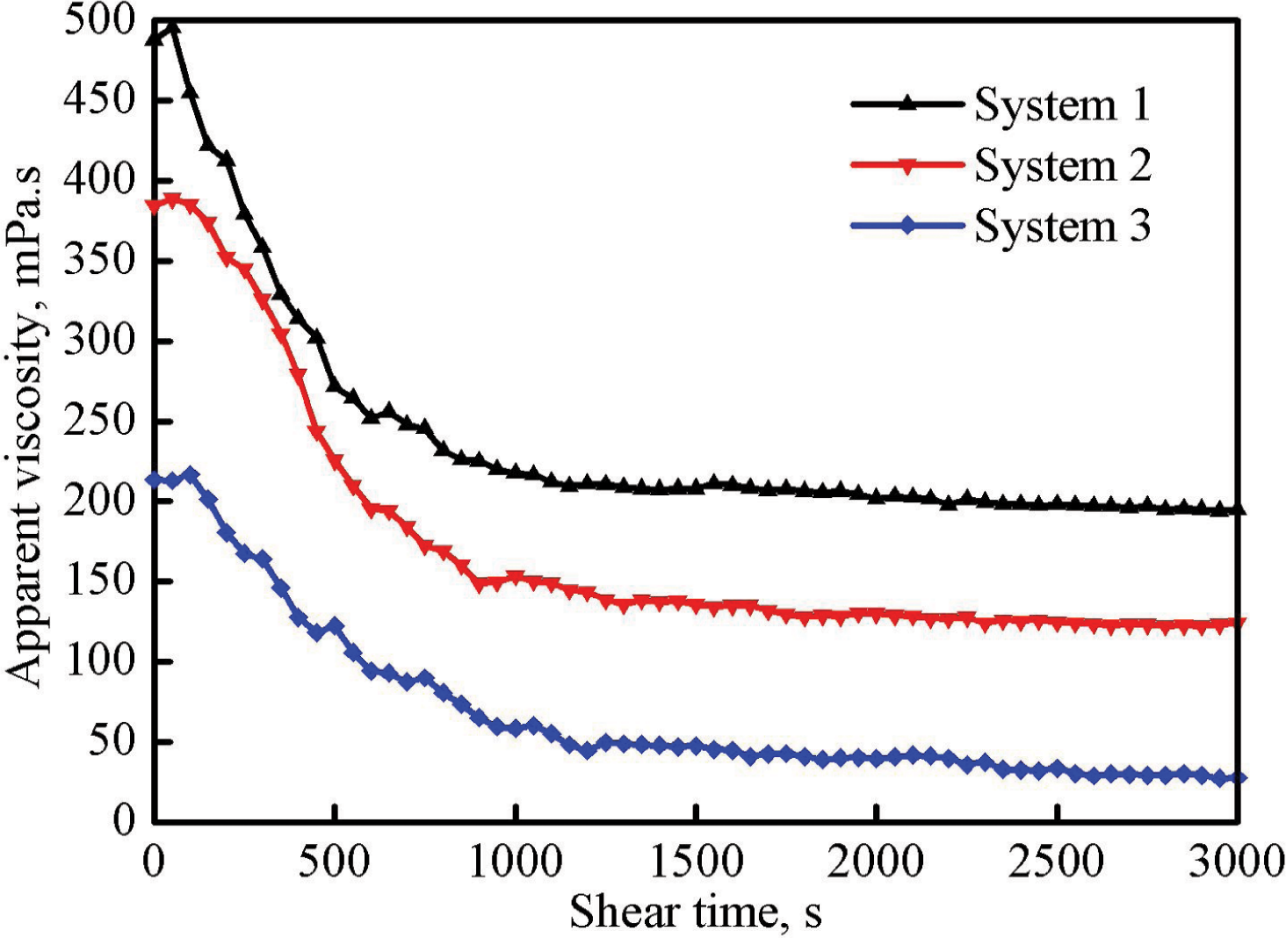
Effect of shear time on the apparent viscosity of three fracturing fluid systems.

Fig 7 shows the influence of shear time on the apparent viscosity of three gelled fracturing fluid systems with a shear rate of 170 1/s. The initial apparent viscosities of systems 1, 2 and 3 were 488.2, 384.6 and 213.6 mPa·s, respectively. The viscosity curve of each system can be divided into three segments. When the shear time was shorter than 50 s, all three fracturing fluids showed a better shear tolerance and their viscosities slightly increased; with the extension of the shear time from 100 to 500 s, a sharp drop in viscosity occurred because of the severe damage of the gel network structure; with a further extension of the shear time, especially when the shear time exceeded 1000 s, all the viscosities of three systems slightly changed and leveled off at 197.1, 123.6 and 28.6 mPa·s, respectively.

The Ostwald–de Waele or power law model was applied to express the shear tolerance of each system, as shown in the Eq (3) [16]:

$$\eta =k\gamma^{n-1}$$ (3)

where *η* is the apparent viscosity in mPa·s, *k* is the consistency index in mPa·s^n^, *γ* is the shear rate in s^-1^ and *n* is the fluid behavior index.

Eq (3) can be modified into the form of the relationship between the natural logarithm of *η* and the natural logarithm of *γ*:

lnη=lnk+(n−1)lnγ (4)

**Fig 8:**
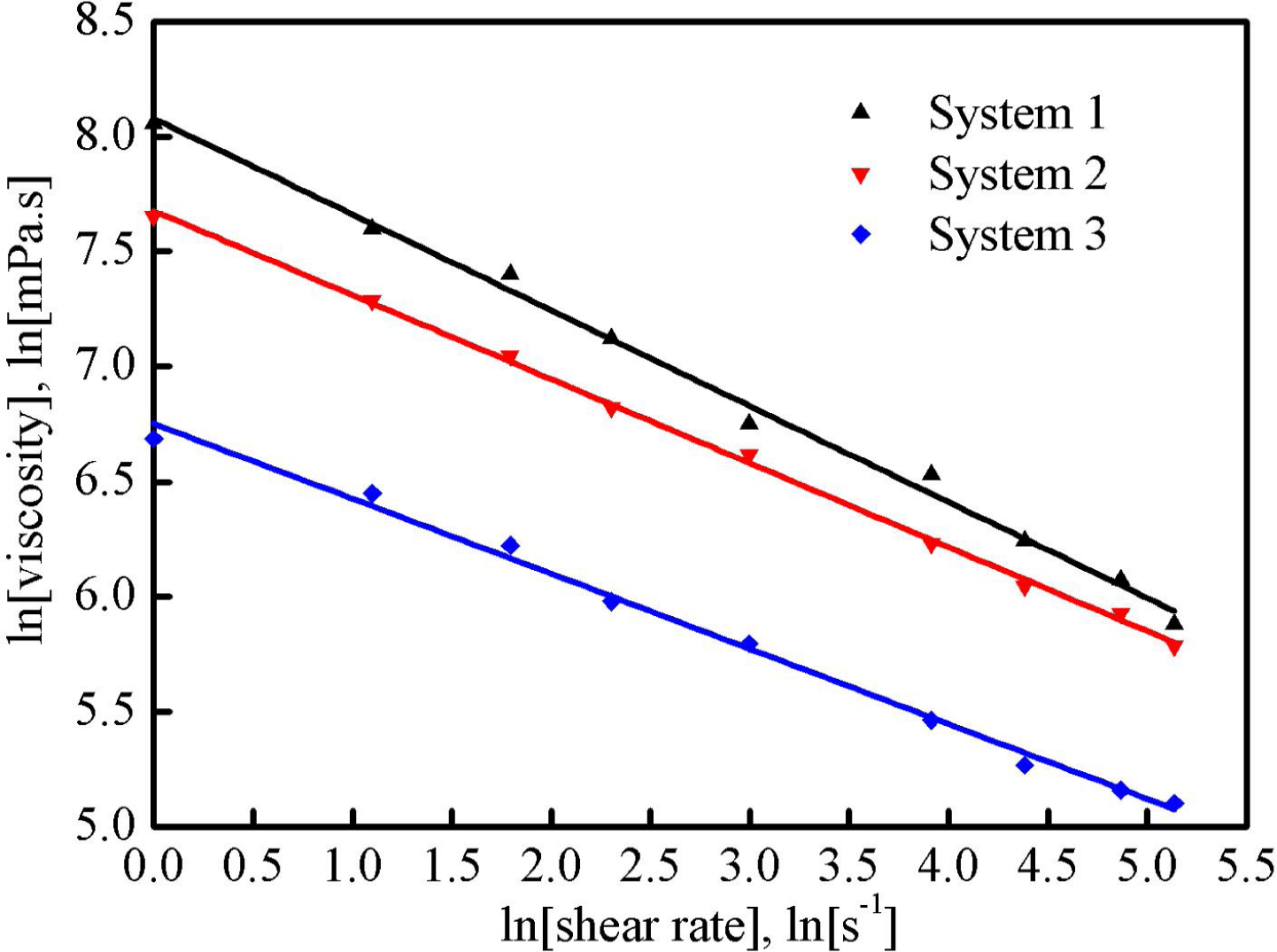
Relationship between natural logarithm values of the shear rate and the apparent viscosity of three fracturing fluid systems.

The effect of shear rate (in the range of 1 to 170 1/s) on the apparent viscosity of each gelled system was studied and the relation between ln *η* and ln *γ* was show in Fig 8. It proves a linear relationship between ln *η* and ln *γ*, and it is consistent with the expression shown in the Eq (4). Further, the Eq (4) can be shown as Eqs (5)–(7) to describe the linear relationship for three systems:

$$\text{system}\;1:\;\ln\eta_{1}=8.0783-0.4168\;\ln\gamma_{1}\;R^{2}=0.9950$$ (5)

$$\text{system}\;2:\;\text{ln}\;\eta_{2}=7.6765-0.3653\;\text{ln}\;\gamma_{2}\;R^{2}=0.9986$$ (6)

$$\text{system}\;3:\;\text{ln}\;\eta_{3}=6.7524-0.3264\;\text{ln}\;\gamma_{3}\;R^{2}=0.9945$$ (7)

The consistency index *k* and the fluid behavior index *n* of each system were calculated using the slope and the intercept in Eqs (5)–(7) and listed in Table 2. *k* represents the viscosifying capacity: the larger the *k* is, the stronger the viscosifying capacity shows. As shown in Table 2, the value of *k* decreased with the increase of the number of regeneration, and a high viscosity was always accompanied by a large value of *k*. *n* represents the flow behavior of a fluid. When *n* is lower than 1, the fluid is pseudoplastic with a characteristic of shear thinning, i.e., the apparent viscosity of this kind of fluid decreased as the shear rate increased. The *n* value of system 1 was 0.5832, it slowly increased to 0.6736 for system 3. It demonstrates that the fresh fracturing fluid was more sensitive to the change of shear rate and it was consistent with the results of microscopic structure and shear stability analyses, i.e., system 1 with a three-dimension network structure was more sensitive to the shear degradation.

**Table 2:** Consistency index and fluid behavior index of three fracturing fluid systems.

No.	*k*, mPa·s^n^	*n*
System 1	3223.75	0.5832
System 2	2157.06	0.6347
System 3	856.11	0.6736

#### Thermal stability evaluation

**Fig 9:**
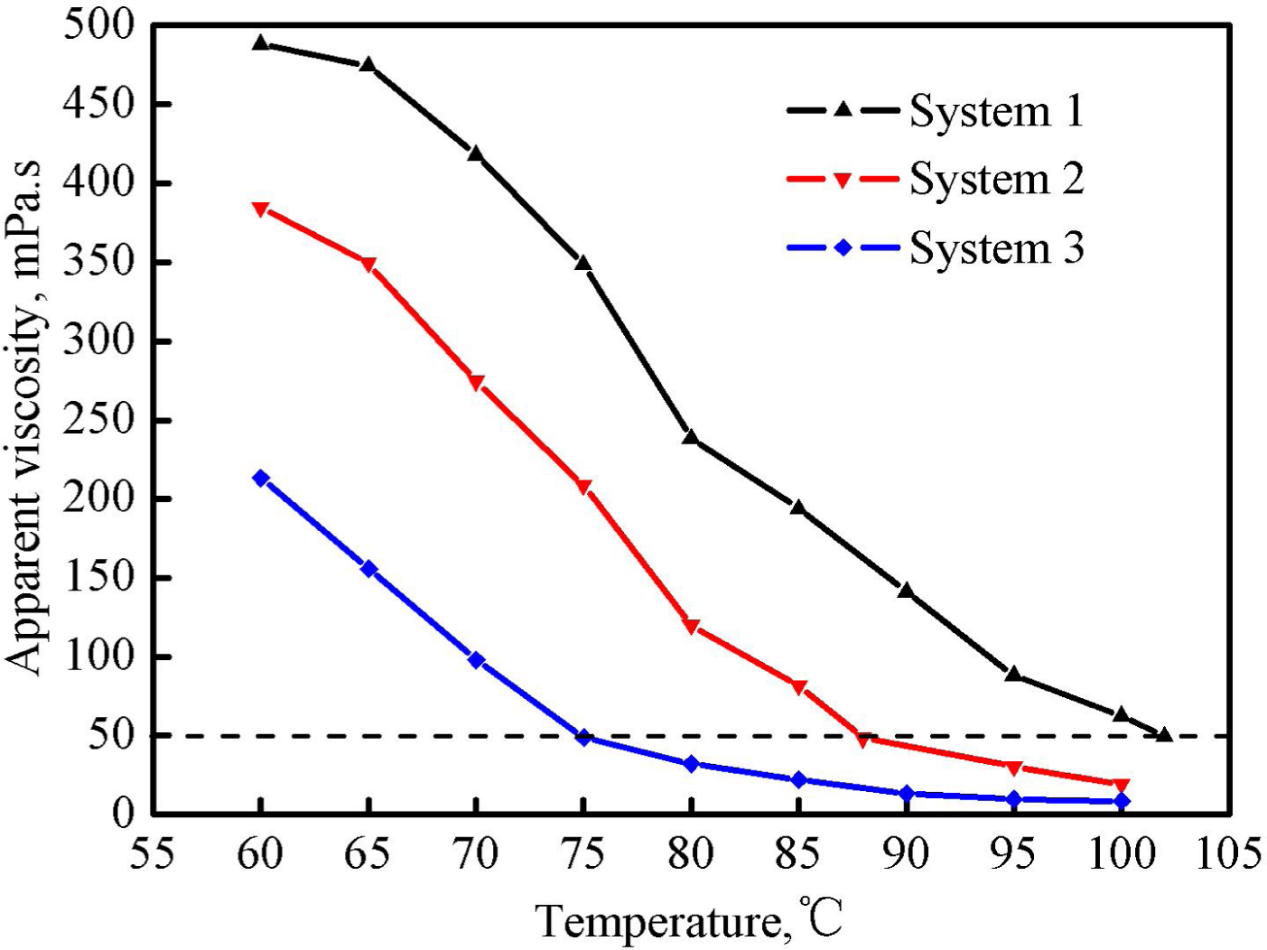
Effect of temperature on the apparent viscosity of three fracturing fluid systems.

Thermal tolerance is an important parameter for the evaluation of a fracturing fluid. There is no doubt that a fracturing fluid with a good thermal stability has relatively wide application [17,18]. Fig 9 shows the effect of temperature on the apparent viscosities of three fracturing fluid systems when the shear rate was 170 1/s. It illustrates that the apparent viscosity of each system was reduced with the increase of temperature. Each viscosity curve can be separated into two segments according to the slope of sub-curves. However, the cross-points at which two curve segments cross with each other in three systems were different and they occurred at 65°C (system1), 88°C(system 2) and 75°C(system 3), respectively.

For system 1 which was without regeneration, the initial viscosity at 60°C was 488.2 mPa·s; with the increase of temperature to 65°C, the viscosity changed slightly (474.5 mPa·s). When the temperature was over 65°C, the viscosity greatly decreased and it was down to about 50 mPa·s at 102°C. In the present study, the temperature at which the apparent viscosity of the fracturing fluid decreased to 50 mPa·s was defined as the critical temperature. For system 2 and 3, the temperatures corresponding to the cross-points were same with the critical temperatures and they were 88°C(system 2) and 75°C(system 3), respectively. Data indicate that the thermal stability of the fracturing fluid was weakened with the increase of the number of regeneration. The high degree of the cross-linking reaction between PVOH and OBT of system 1 was responsible for its better thermal stability.

#### Viscoelasticity evaluation

The viscoelasticity of the gelled fracturing fluid can be described using the storage modulus G' and the loss modulus G'' [19]. G' represents the energy storage ability of a material in the reversible deformation process. The higher the storage modulus is, the better the elasticity becomes. G'' represents the energy loss of a material in the irreversible deformation process and a material with better viscosity often holds a bigger loss modulus [20]. If the value of G' is higher than that of G'', the elasticity holds the dominant position; otherwise, the viscosity takes the advantage.

**Fig 10:**
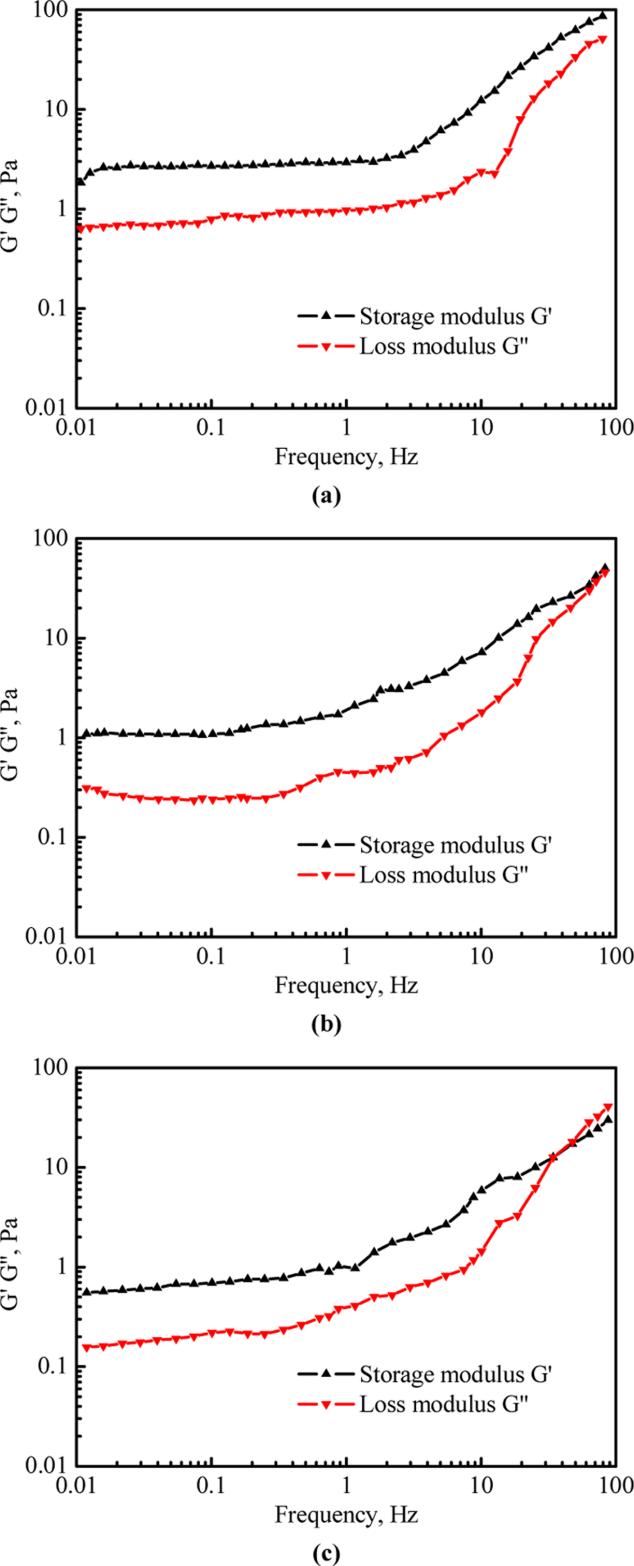
Storage modulus G' and loss modulus G'' of three fracturing fluid systems vs. scan frequency. (a) System 1; (b) system 2; (c) system 3.

Fig 10(A), 10(B) and 10(C) respectively show evolutions of G' and G'' of systems 1, 2 and 3 with the variation of scan frequency. For each system, G' and G'' changed slightly when the scan frequency was lower than 1 Hz, and both of them rose after the scan frequency exceeded 1 Hz. For system 1, G' was always greater than G'' in the range of scan frequency and it indicates that the elasticity always takes the advantage due to the three-dimensional network structure [21]. The difference between the elasticity and the viscosity of the regenerated fracturing fluid changed when the fracturing fluid was regenerated once or twice. For system 2, the G' value was close to G'' value when the scan frequency was over 46 Hz; for system 3, a cross-point of G' and G'' curves occurred when the scan frequency exceeded 34 Hz. The damage of the gel structure in the regeneration process for systems 2 and 3 results in the weakened increment of the elasticity.

Fig 10 also shows that both the values of G' and G' are stable at low frequencies but increase at high frequencies. The three-dimensional network structure consists of lots of cross-linked molecular chains inside the gelled fracturing fluid. When the scan frequency was high enough, the molecular chains were obviously stretched and the in situ microfibrillar structure which could greatly enhance the dependence of G' on the scan frequency emerged to strengthen the elasticity [22]. Moreover, part of the microfibrillar structure was easily destroyed by the shear force when the scan frequency was high enough, and then the dependence of the loss modulus G'' on the scan frequency was enhanced and the G'' value increases.

The loss tangent tanδ was employed to further explore the change of the relationship between the elasticity and the viscosity of three fracturing fluids, as shown in the Eq (8), and the increasing value of tan*δ* indicates that the loss modulus holds the dominant position in the deformation process [23,24].

tanδ=G''/G' (8)

where tan*δ* is the loss tangent, G' is the storage modulus in Pa, and G'' is the loss modulus in Pa.

**Fig 11:**
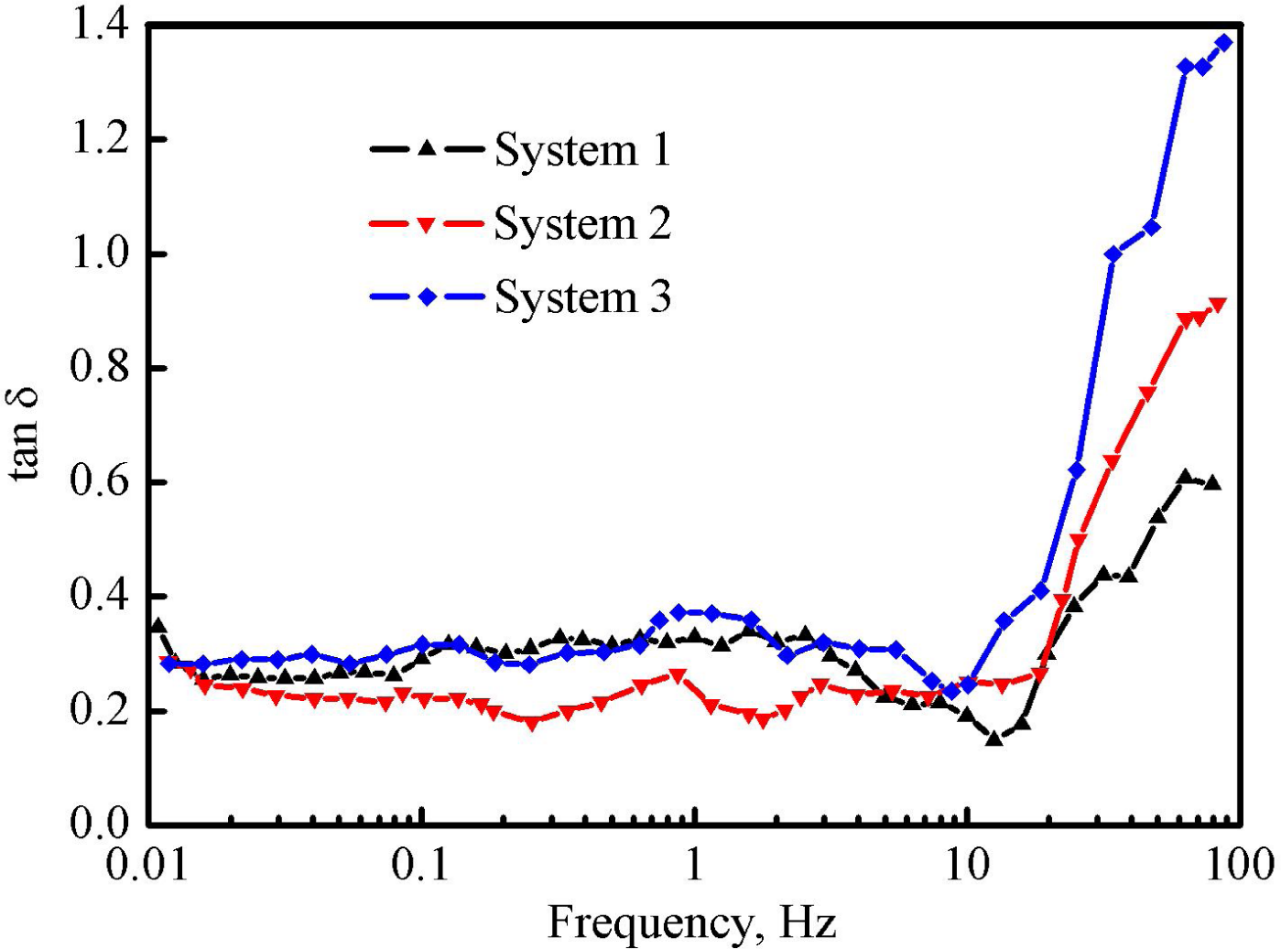
Relationship between the loss tangent and the scan frequency of three fracturing fluid systems.

Fig 11 shows the change in the relationship between the loss tangent and the scan frequency of three fracturing fluid systems. When the scan frequency was lower than 10 Hz, tan*δ* values of three systems were in the range of 0.2–0.4 and slightly changed; with a further increase of the scan frequency, tan*δ* sharply increased because of the great increment of G'' than that of G'. Moreover, the differences among three tan*δ* curves were gradually enlarged after the scan frequency exceeded 10 Hz. Final tan*δ* values of systems 1, 2 and 3 were 0.59, 0.91 and 1.37, respectively.

The analyses of G', G'' and tan*δ* indicate that the viscosity property of the regenerated fracturing fluid gradually took the advantage compared with the elasticity property, and it reflects a fact that the network structure of the gelled fracturing fluid was destroyed after regeneration.

### Proppant Carrying Capacity

**Fig 12:**
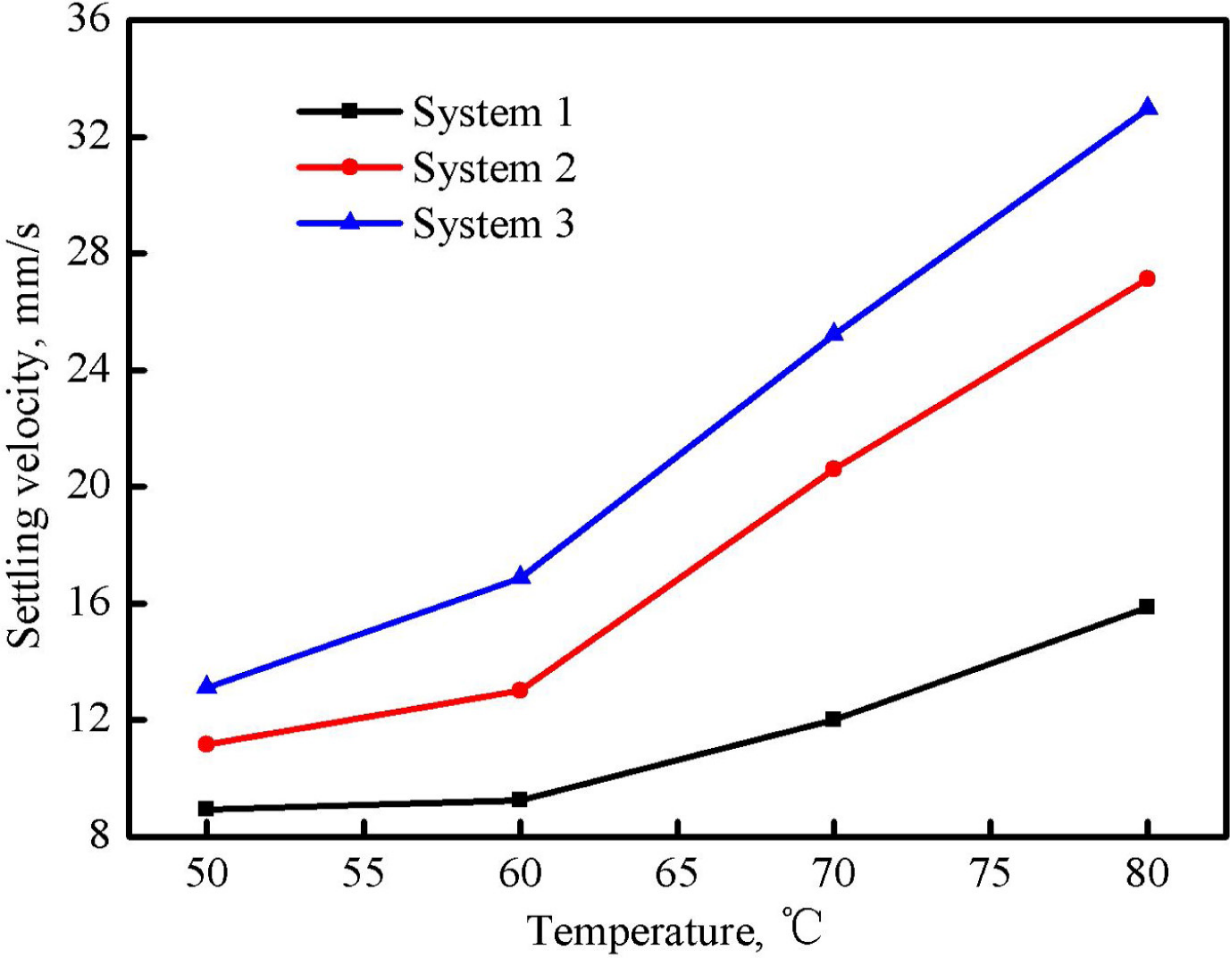
Effect of temperature on the proppant settling velocity of three fracturing fluid systems (proppant ratio was 10%).

Fig 12 illustrates the effect of temperature on the proppant settling velocity when the proppant ratio in three fracturing fluid systems was 10%. It shows that the proppant settling velocity was accelerated with the increase of the temperature. The increment of the settling velocity was small at the beginning but became larger and larger after the temperature exceeded 60°C. By comparing the settling velocities of three systems, the proppant carrying capacity of system 1 was more favorable and system 2 took the second place. The main reason is that the increase of the temperature reduces both the apparent viscosity and the elasticity of fracturing fluids, and thus reduces the proppant carrying capacity [25].

**Fig 13:**
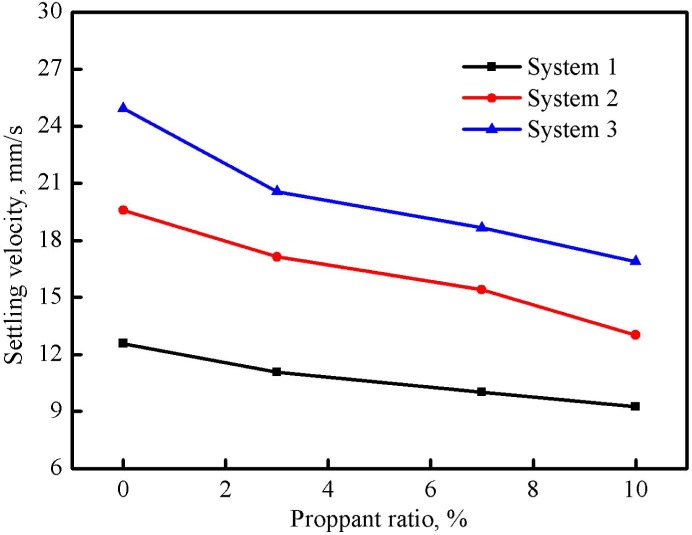
Effect of proppant ratio on the proppant settling velocity of three fracturing fluid systems (temperature was 60°C).

The influence of the proppant ratio on the proppant settling velocity of three fracturing fluid systems was also investigated at 60°C and the results are shown in Fig 13. The proppant settling velocity was gradually decelerated with the increase of the proppant ratio from single particle to 10%. Two explanations account for this phenomenon: firstly, the proppant settlement is always accompanied by the fluid reverse-flow which will become greater and greater with the increase of the proppant ratio and reduce the settling velocity [26]; secondly, the mutual disturbance among proppant particles also decelerates the proppant settling velocity.

### Core Permeability Damage

**Fig 14:**
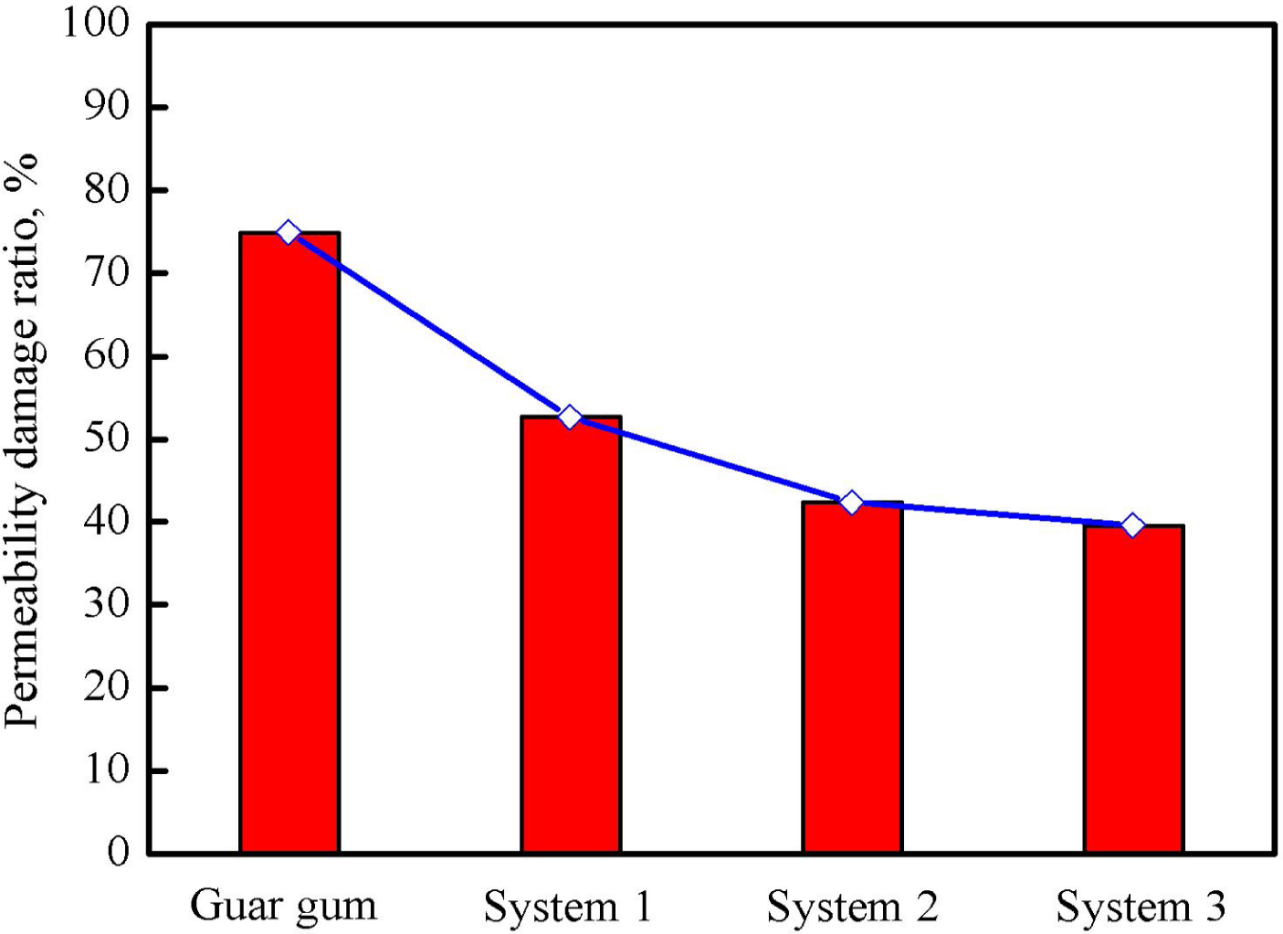
Core permeability damage ratios of four fracturing fluid systems after gel breaking: guar gum (0.35%) system and PVOH/OBT systems 1, 2 and 3.

**Fig 15:**
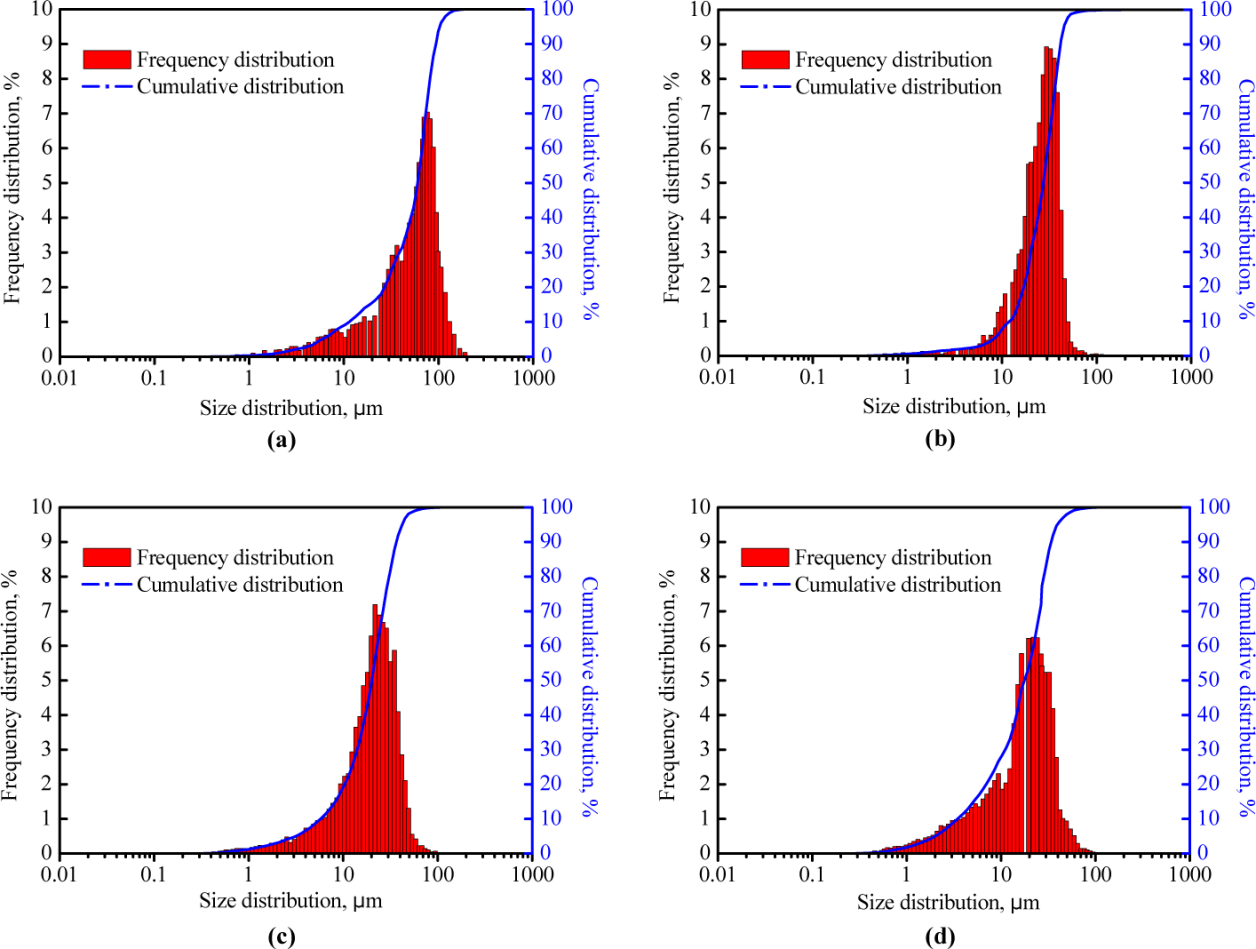
Particle size distributions of four fracturing fluid systems after gel breaking. (a) 0.35% guar gum system; (b) system 1; (c) system 2; (d) system 3.

The degree of the core permeability damage of three fracturing fluid systems after gel breaking was measured according to the procedure described in the section 2.5; meanwhile, the common guar gum (0.35%) fracturing fluid was also used as a contrast. Fig 14 shows that the permeability damage ratio caused by the guar gum fracturing fluid was 74.89%; for systems 1, 2 and 3, they were 52.64, 42.33 and 39.57%, respectively. Data indicate that guar gum system caused the most serious damage to the core. In general, the residual fracturing fluid with dispersed gel particles after incompletely gel breaking takes the main responsibility for the damage of the fracture conductivity and the adjacent matrix permeability [27,28]. Therefore, particle size distributions of guar gum fluid and systems 1–3 after gel breaking were explored with a holding time of 24 hours, and the frequency and cumulative distribution curves are shown in Fig 15(A), 15(B), 15(C) and 15(D).

For system 1, the median size of more than 80% of the gel particles was in the range of 10–45 μm, and they were in the range of 5–40 μm for system 2 and 4–40 μm for system 3, respectively. Data in Table 3 show that the median size of the fracturing fluid decreased with the increase of the regeneration number of times. A possible explanation is that the broken cross-linking sites of PVOH and OBT remain outside of gel particles after gel breaking and they can cross-link again to form a new gel structure in the regeneration process; with the increasing number of gel regeneration, more cross-linking sites are broken and trapped into gel particles, and it results in a weak degree of cross-linking reaction and small gel particles in the next regeneration process. Therefore, the particle sizes of systems 2 and 3 are smaller than that of system 1. In addition, the median size of the guar gum fracturing fluid after gel breaking was 73.855 μm. The median sizes of the broken guar gum reported by Guo et al [29]. and Liu et al [30]. were 89.09 and 72.45 μm, respectively. Results indicate that less permeability damage will be caused by the PVOH/OBT fracturing fluid compared with that caused by the common guar gum fracturing fluid.

**Table 3:** Median size of three fracturing fluid systems after gel breaking.

No.	0.35% Guar gum	System 1	System 2	System 3
Median size, μm	73.855	27.582	19.971	18.639

## Conclusions

1. A regenerable polyvinyl alcohol/organic boron fracturing fluid system with 1.6 wt% PVOH and 1.2 wt% OBT is optimized, and the suitable pH range for the formation of the PVOH/OBT fracturing fluid when its apparent viscosity exceeds 400 mPa·s is 8.5–10.
2. The apparent viscosity of the fracturing fluid decreases with the regeneration number of times increasing. The viscosity of system 1 which is without regeneration and owns a three-dimension network structure is more sensitive to the shear rate compared with that of system 2 or system 3.
3. The thermal stability of the fracturing fluid became weaker with the increase of the regeneration number of times. When the fracturing fluid is without regeneration, its elasticity property takes the advantage, but the viscosity property gradually occupies the advantage when the fracturing fluid is regenerated once or twice.
4. Both the regeneration process and the experimental temperature can accelerate the proppant settling velocity, but the proppant settling velocity is decelerated with the increase of the proppant ratio in the fracturing fluid.
5. Less permeability damage is caused by the PVOH/OBT fracturing fluid compared with that caused by the commom guar gum fracturing fluid after gel breaking.
